# Treatment patterns and healthcare resource use among veterans initiating medication for incident moderate‐to‐severe alcohol use disorder

**DOI:** 10.1111/ajad.70036

**Published:** 2025-05-12

**Authors:** Regina Grebla, Teresa L. Kauf, Angela Lax, Erin E. Cook, Yilu Lin, Jieruo Liu, Shuqian Liu, Amy K. O'Sullivan, Lizheng Shi, Sherry Shi, Maria A. Sullivan, Elyse Swallow, Katie Witkiewitz, Karen Drexler

**Affiliations:** ^1^ Alkermes, Inc. Waltham Massachusetts USA; ^2^ Analysis Group, Inc. Boston Massachusetts USA; ^3^ Southeast Louisiana Veterans Health Care System New Orleans Louisiana USA; ^4^ Tulane University New Orleans Louisiana USA; ^5^ Groupe d'Analyse Montréal Quebec Canada; ^6^ Columbia University New York New York USA; ^7^ University of New Mexico Albuquerque New Mexico USA; ^8^ Emory University School of Medicine Atlanta Georgia USA

## Abstract

**Background and Objectives:**

Several medications for alcohol use disorder (MAUDs) are recommended to treat alcohol use disorder (AUD) in the Veterans Affairs (VA) guidelines. This study descriptively characterized treatment patterns and healthcare resource utilization (HCRU) among VA patients with AUD treated with VA‐recommended MAUDs.

**Methods:**

Veterans Health Administration data (VHA; 08/01/2013–11/30/2019) were used to identify 31,384 adults aged ≥18 years with AUD who initiated disulfiram (*n* = 2115), acamprosate (*n* = 3756), oral naltrexone (*n* = 25,082), or extended‐release naltrexone (XR‐NTX; *n* = 431) following AUD diagnosis. Study measures, stratified by medication received, included treatment adherence (proportion of days covered), discontinuation, and HCRU over 1 year.

**Results:**

Mean time to treatment discontinuation was high for all MAUDs but longest for XR‐NTX (92 vs. 55–59 days; all *p* < .001). Relative to the year preceding AUD diagnosis, treatment with MAUDs was associated with fewer hospitalizations (XR‐NTX: 0.48 vs. 0.42; oral naltrexone: 0.58 vs. 0.47; acamprosate: 0.67 vs. 0.60; disulfiram: 0.63 vs. 0.57) and more outpatient visits per patient (XR‐NTX: 20.0 vs. 36.0; oral naltrexone: 19.0 vs. 30.0; acamprosate: 19.0 vs. 31.0; disulfiram: 17.0 vs. 29.0).

**Conclusion:**

Among veterans with AUD, this descriptive analysis found that MAUD use was associated with reduced hospitalizations, and XR‐NTX was associated with a longer treatment duration versus oral MAUDs.

**Scientific Significance:**

This real‐world study is among the first to describe clinical characteristics, treatment patterns, and HCRU in VHA patients who initiated MAUDs when all MAUDs were included in the VHA formulary.

## INTRODUCTION

Alcohol use disorder (AUD) is a psychiatric condition characterized by continued, persistent use of alcohol despite detrimental social, occupational, and health‐related consequences.^1^ In 2020, approximately 27.6 million adults in the United States (US) had AUD.^2^

In addition to behavioral counseling, several pharmacotherapies have been approved by the US Food and Drug Administration (FDA) to treat Diagnostic and Statistical Manual of Mental Disorders, 4th edition (DSM‐IV) alcohol dependence (AD), which corresponds to DSM‐5 moderate‐to‐severe AUD.^3^, ^4^, ^5^ These include oral medications, disulfiram,^6^ acamprosate calcium (acamprosate),^7^ and oral naltrexone,^8^ and extended‐release naltrexone (XR‐NTX), which is administered as a single intramuscular injection once a month.^9^ Although medications for AUD (MAUDs) are effective, access to treatment, patient adherence, and length of treatment may affect clinical outcomes.^10^, ^11^, ^12^, ^13^ Since 2014, the Veterans Health Administration (VHA) has provided all FDA‐approved MAUDs on formulary^14^ and has implemented nationwide training initiatives to increase access to treatment.^12^, ^13^, ^15^ To improve MAUD treatment persistence, guidelines from the US Department of Veterans Affairs (VA) and Department of Defense (DoD) recommend XR‐NTX be considered when treatment adherence is a concern.^9^, ^16^, ^17^

Real‐world evidence regarding the use and health outcomes of XR‐NTX relative to other MAUDs is limited. This study aimed to descriptively summarize treatment patterns and healthcare resource utilization (HCRU) among US veterans with moderate‐to‐severe AUD who initiated treatment with MAUDs.

## METHODS

### Data source

This retrospective, observational study used data from the VHA (August 1, 2013, to November 30, 2019). Data were accessed through the VHA's Corporate Data Warehouse (CDW), which comprises administrative claims and linked electronic medical records for roughly 9 million beneficiaries annually. The CDW, which has been described previously,^18^ contains information on all outpatient visits, hospital stays, treatments administered, dispensed prescriptions, and laboratory results, in addition to billing and benefits information, for healthcare encounters that occur at VHA facilities. This study was approved by the Southeast Louisiana Veterans Health Care System Research & Development Committee on September 22, 2020.

### Study design and sample selection

**Figure 1:**
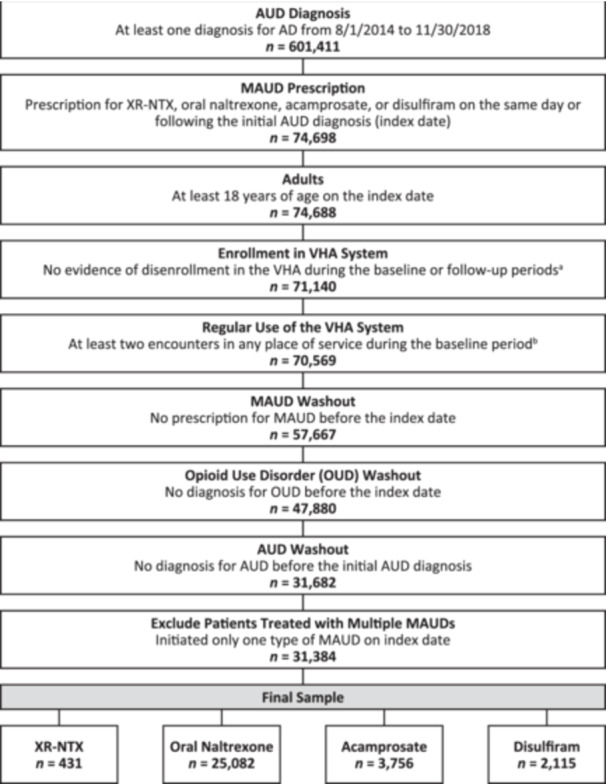
Sample selection by index treatment. AD, alcohol dependence; AUD, alcohol use disorder; MAUD, medications for alcohol use disorder; OUD, opioid use disorder; VHA, Veterans Health Administration; XR‐NTX, extended‐release naltrexone. ^a^Unlike other health claims databases, the Veterans Affairs Corporate Data Warehouse does not have an eligibility file. However, there is a variable for disenrollment, which was used to ensure patients are currently enrolled. ^b^As patients are automatically enrolled in the VHA upon return from military service, patients may or may not use the VHA services for their medical care. To identify patients with regular use of the VHA, we required at least two encounters during the baseline period.

To be included, patients were required to have at least one medical record containing an International Classification of Diseases Ninth/Tenth Revision, Clinical Modification (ICD‐9/10‐CM) diagnosis code for AD (ICD‐9‐CM: 303; ICD‐10‐CM: F10.2) in any place of service from August 1, 2014 to November 30, 2018; for patients with multiple diagnoses for AD, the first was defined as the initial moderate‐to‐severe AUD diagnosis. Patients also were required to have at least one record for a MAUD on or after the AUD diagnosis; the date of this record was defined as the index date. The 1‐year period preceding the index date was defined as the baseline period, and the 1‐year period after the index date was defined as the follow‐up period (Supplementary Information S1: Figure 1).

Patient cohorts were based on the index MAUD. Patients were required to be ≥18 years of age on the index date and have no evidence of disenrollment in the VHA during the baseline and follow‐up periods. To ensure that patients regularly used the VHA, at least two encounters during the baseline period were required. Patients who received a MAUD any time before the index date, or had evidence of multiple MAUDs on the index date, were excluded. Patients with prior diagnoses for opioid use disorder were also excluded, so it was clear that the use of a MAUD was for AUD.

### Study measures and outcomes

Patient demographics were assessed on the index date. Clinical characteristics and all‐cause HCRU were assessed during the baseline period. Additional study measures included the index treatment setting and time from initial AUD diagnosis to initiation of the index MAUD.

Outcomes evaluated during the follow‐up period included treatment patterns and all‐cause HCRU. Specific treatment patterns that were assessed included adherence (proportion of days covered [PDC]), discontinuation, MAUD initiation post‐index treatment discontinuation, and concurrent treatment used during the index treatment. To compute the PDC, the number of days that a patient was covered by a MAUD in the 1‐year follow‐up period was divided by 365.

Treatment discontinuation in our study was defined as no subsequent record of the index treatment in the 45‐day period following the previous treatment record, beginning from the date of the prior record/fill date. Given that oral MAUDs are typically provided in a 30‐day supply (including mail‐order) and XR‐NTX is administered approximately every 28 days, this definition allows for an approximately 2‐week grace period, which is similar to that in other studies.^19^, ^20^, ^21^ The final date of the days of supply (i.e., the number of days of medication the patient received in one prescription) was assigned as the date of discontinuation.

### Statistical analyses

Study measures and outcomes were summarized using descriptive statistics. Continuous variables were described using means, standard deviations, and medians; categorical variables were described using counts and percentages. Pairwise comparisons of XR‐NTX with individual MAUDs (oral naltrexone, acamprosate, and disulfiram) were conducted using *t*‐tests for continuous variables and chi‐square tests for categorical variables. HCRU assessed during the baseline and follow‐up periods within each MAUD cohort were summarized descriptively. There was no effort to control for multiple comparisons in these analyses. No formal statistical testing between baseline and follow‐up HCRU was conducted.

## RESULTS

### Patient and clinical characteristics

Of the 601,411 patients covered by the VHA who were diagnosed with moderate‐to‐severe AUD between August 1, 2014 and November 30, 2018, 31,384 met all inclusion criteria for the study. The final study sample included 431 patients in the XR‐NTX cohort, 25,082 patients in the oral naltrexone cohort, 3756 patients in the acamprosate cohort, and 2115 patients in the disulfiram cohort (Figure 1).

Patient characteristics across cohorts are summarized in Table 1. Across all cohorts, there were higher proportions of patients who initiated MAUD treatment in later years (range: 4%–7% in 2014, 20%–30% in 2018; 12.4% across the study period). Overall, the mean age of patients in the XR‐NTX cohort (46.0 years) was lower compared with the other MAUDs (47.9 years for oral naltrexone [*p* < .01], 50.5 years for acamprosate [*p* < .001], and 47.1 years for disulfiram [*p* = .10]). Across all cohorts, most patients were male (range: 90.3%–92.3%). Additionally, most patients in each cohort were White, with a significantly higher proportion in the XR‐NTX cohort (78.7%) compared with the oral naltrexone cohort (65.5%, *p* < .001) and the acamprosate cohort (69.2%, *p* < .001). Employment status was similar across the treatment cohorts, with 34.6% to 37.7% of patients employed.

**Table 1:** Patient demographics and clinical characteristics.

	XR‐NTX	Oral naltrexone		Acamprosate		Disulfiram		Total
	*N* = 431	*N* = 25,082	*p*‐value^a^	*N* = 3756	*p*‐value^a^	*N* = 2115	*p*‐value^a^	*N* = 31,384
**Demographics**								
Age at index date (years),^b^ mean ± SD [median]	46.0 ± 12.8 [46]	47.9 ± 13.5 [48]	**<.01**	50.5 ± 13.1 [51]	**<.001**	47.1 ± 13.5 [47]	.104	48.1 ± 13.5 [48]
Year of index date, *n* (%)								
2014	22 (5.1)	1011 (4.0)	.262	180 (4.8)	.775	140 (6.6)	.240	1353 (4.3)
2015	79 (18.3)	4050 (16.1)	.223	700 (18.6)	.877	477 (22.6)	.053	5306 (16.9)
2016	78 (18.1)	5493 (21.9)	.058	844 (22.5)	**<.05**	570 (27.0)	**<.01**	6985 (22.3)
2017	122 (28.3)	7084 (28.2)	.977	1002 (26.7)	.470	498 (23.5)	**<.05**	8706 (27.7)
2018	130 (30.2)	7444 (29.7)	.828	1030 (27.4)	.229	430 (20.3)	**<.001**	9034 (28.8)
Male, *n* (%)	389 (90.3)	22,752 (90.7)	.747	3458 (92.1)	.192	1952 (92.3)	.156	28,551 (91.0)
Race, *n* (%)								
White	339 (78.7)	16,419 (65.5)	**<.001**	2600 (69.2)	**<.001**	1649 (78.0)	.753	21,007 (66.9)
Black	58 (13.5)	6300 (25.1)	**<.001**	849 (22.6)	**<.001**	302 (14.3)	.655	7509 (23.9)
Asian, Pacific Islander, or Native American	12 (2.8)	916 (3.7)	.340	132 (3.5)	.431	60 (2.8)	.952	1,120 (3.6)
Unknown	22 (5.1)	1447 (5.8)	.557	175 (4.7)	.679	104 (4.9)	.870	1748 (5.6)
Employment status, *n* (%)								
Employed	160 (37.1)	9233 (36.8)	.894	1301 (34.6)	.305	797 (37.7)	.827	11,491 (36.6)
Active military duty	9 (2.1)	272 (1.1)	.058	45 (1.2)	.121	19 (0.9)	**<.05**	345 (1.1)
Not employed	202 (46.9)	11,524 (45.9)	.703	1778 (47.3)	.853	959 (45.3)	.562	14,463 (46.1)
Retired	29 (6.7)	2003 (8.0)	.339	406 (10.8)	**<.01**	175 (8.3)	.281	2,613 (8.3)
Unknown	40 (9.3)	2,322 (9.3)	.987	271 (7.2)	.121	184 (8.7)	.698	2817 (9.0)
**Comorbidities, *n* (%)** c								
Anxiety disorder	50 (11.6)	3278 (13.1)	.370	460 (12.2)	.698	366 (17.3)	**<.01**	4154 (13.2)
Sleep disorder	116 (26.9)	6461 (25.8)	.587	973 (25.9)	.651	480 (22.7)	.059	8030 (25.6)
Posttraumatic stress syndrome	192 (44.5)	10,913 (43.5)	.666	1490 (39.7)	.050	895 (42.3)	.394	13,490 (43.0)
Major depressive disorder	235 (54.5)	13,602 (54.2)	.903	1975 (52.6)	.444	1048 (49.6)	.060	16,860 (53.7)
Schizophrenia	14 (3.2)	481 (1.9)	**<.05**	79 (2.1)	.127	28 (1.3)	**<.01**	602 (1.9)
Bipolar disorder	44 (10.2)	2037 (8.1)	.116	286 (7.6)	.058	170 (8.0)	.139	2537 (8.1)
Chronic pain	18 (4.2)	1510 (6.0)	.110	352 (9.4)	**<.01**	146 (6.9)	**<.05**	2026 (6.5)
Hypertension	157 (36.4)	9908 (39.5)	.195	1817 (48.4)	**<.001**	783 (37.0)	.816	12,665 (40.4)
Diabetes	47 (10.9)	2520 (10.0)	.557	476 (12.7)	.293	169 (8.0)	**<.05**	3212 (10.2)
CCI, mean ± SD [median]	0.5 ± 1.0 [0]	0.5 ± 1.1 [0]	.778	0.9 ± 1.4 [0]	**<.001**	0.5 ± 1.1 [0]	.977	0.5 ± 1.1 [0]
**Substance use disorders** d								
Any substance use disorder, *n* (%)	233 (54.1)	12,310 (49.1)	**<.05**	1864 (49.6)	.081	1012 (47.8)	**<.05**	15,419 (49.1)
Cannabis	63 (14.6)	4275 (17.0)	.184	604 (16.1)	.432	284 (13.4)	.512	5226 (16.7)
Cocaine	43 (10.0)	2957 (11.8)	.247	350 (9.3)	.657	195 (9.2)	.623	3545 (11.3)
Nicotine	151 (35.0)	7940 (31.7)	.135	1292 (34.4)	.792	712 (33.7)	.584	10,095 (32.2)
Other psychoactive substance	73 (16.9)	3626 (14.5)	.147	495 (13.2)	**<.05**	293 (13.9)	.096	45 (0.1)
**Psychiatric medications** e								
Number of unique psychiatric medications, mean ± SD [median]	2.1 ± 1.9 [2]	2.0 ± 1.9 [2]	.156	2.2 ± 1.9 [2]	.654	2.1 ± 1.9 [2]	.582	2.0 ± 1.9 [2]
**Duration between first AUD diagnosis and index date (days), mean ± SD [median]**	233.4 ± 331.5 [71]	257.0 ± 346.4 [76]	.161	264.6 ± 345.2 [87]	.075	228.6 ± 317.6 [71]	.773	255.7 ± 344.3 [77]

*Note*: Bold values indicate a statistically significant difference defined by *p* < .05.

Abbreviations: AUD, alcohol use disorder; CCI, Charlson comorbidity index; SD, standard deviation; XR‐NTX, extended‐release naltrexone.

^a^ Statistical comparisons were conducted between XR‐NTX and other MAUD cohorts using *t*‐tests for continuous variables and chi‐square tests for categorical variables. A *p*‐value <.05 was considered statistically significant.

^b^ The index date was defined as the date of the patient's first prescription for XR‐NTX, oral naltrexone, acamprosate, or disulfiram.

^c^ Comorbidities were defined as at least one claim at any place of service with an associated diagnosis code.

^d^ Substance use was defined as claims in any place of service with an associated diagnosis code.

^e^ Psychiatric medications include antidepressants, antipsychotics, antianxiety medications, and combination psychotherapeutics, and the mean is calculated among all patients.

Baseline clinical characteristics were generally similar across cohorts (Table 1). Major depressive disorder and hypertension were the most commonly occurring mental health and physical comorbidities overall. There was a significantly higher proportion of patients with schizophrenia at baseline in the XR‐NTX cohort (3.2%) versus the oral naltrexone cohort (1.9%, *p* < .05) and the disulfiram cohort (1.3%, *p* < .01). A higher proportion of patients in the XR‐NTX cohort had a history of any non‐opioid substance use disorder (54.1%) versus the oral naltrexone cohort (49.1%, *p* < .05) and the disulfiram cohort (47.8%, *p* < .05), but were similar to the acamprosate cohort (49.6%, *p* = .08). Patients in each cohort received a median of 2 unique psychiatric medications during baseline. The average time from initial AUD diagnosis to index treatment initiation across all MAUDs was 255.7 days (standard deviation [SD]: 344.4 days, median: 77 days) and ranged from 228.6 [317.6)] to 264.6 [345.2] days across the 4 cohorts.

### Index treatment characteristics and treatment patterns

Table 2 summarizes index treatment characteristics and treatment patterns. Among patients in the XR‐NTX cohort, 90.0% initiated treatment in the outpatient setting, compared with 72.5%, 69.7%, and 71.2% of patients in the oral naltrexone, acamprosate, and disulfiram cohorts, respectively (all *p* < .001). The majority of patients (90.7%–94.9%) did not receive concurrent MAUDs during their index treatment (Table 2). Fewer patients who initiated treatment with XR‐NTX discontinued treatment (94.7%) during follow‐up relative to patients who initiated other MAUDs (range: 99.4%–99.6%; *p* < .001 for all comparisons). The mean time to discontinuation of the index treatment was 91.8 days among the XR‐NTX cohort, 58.7 days among the oral naltrexone cohort (*p* < .001), 58.0 days among the acamprosate cohort (*p* < .001), and 55.1 days among the disulfiram cohort (*p* < .001) (Table 2).

**Table 2:** Index treatment and treatment patterns.

	XR‐NTX	Oral naltrexone		Acamprosate		Disulfiram	
	*N* = 431	*N* = 25,082	*p*‐Value^a^	*N* = 3756	*p*‐Value^a^	*N* = 2115	*p*‐Value^a^
**Index treatment setting, *n* (%)**							
Inpatient	42 (9.7)	6893 (27.5)	**<.001**	1137 (30.3)	**<.001**	609 (28.8)	**<.001**
Outpatient	388 (90.0)	18,189 (72.5)	**<.001**	2619 (69.7)	**<.001**	1506 (71.2)	**<.001**
Emergency department^b^	1 (0.2)	‐	‐	‐	‐	‐	‐
**Concurrent medication use during first‐line treatment** c							
None, *n* (%)	391 (90.7)	23,187 (92.4)	.180	3566 (94.9)	**<.01**	1948 (92.1)	.338
XR‐NTX, *n* (%)	‐	1491 (5.9)	‐	8 (0.2)	‐	8 (0.4)	‐
Oral naltrexone, *n* (%)	31 (7.2)	‐	‐	129 (3.4)	**<.01**	121 (5.7)	.240
Acamprosate, *n* (%)	2 (0.5)	243 (1.0)	.450	‐	‐	14 (0.7)	1.000
Disulfiram, *n* (%)	5 (1.2)	127 (0.5)	.074	25 (0.7)	.228	‐	‐
Multiple MAUDs, *n* (%)	2 (0.5)	34 (0.1)	.123	28 (0.7)	.763	24 (1.1)	.294
**Patients discontinuing index treatment, *n* (%)** d	408 (94.7)	24,937 (99.4)	**<.001**	3741 (99.6)	**<.001**	2103 (99.4)	**<.001**
Days to index treatment discontinuation,^e^^,^ f mean ± SD [median]	91.77 ± 74.25 [62]	58.70 ± 47.20 [33]	**<.001**	57.95 ± 45.99 [34]	**<.001**	55.06 ± 45.48 [31]	**<.001**
**Adherence**							
PDC, mean ± SD [median]^g^	0.34 ± 0.27 [0]	0.29 ± 0.24 [0]	**<.01**	0.26 ± 0.21 [0]	**<.001**	0.29 ± 0.24 [0]	**<.01**

*Note*: Bold values indicate a statistically significant difference defined by *p* < .05.

Abbreviations: MAUD, medications for alcohol use disorder; PDC, proportion days covered; SD, standard deviation; XR‐NTX, extended‐release naltrexone.

^a^ Statistical comparisons were conducted between XR‐NTX and other MAUD cohorts using *t*‐tests for continuous variables and chi‐square tests for categorical variables. A *p*‐value <.05 was considered statistically significant.

^b^ Emergency department setting is identifiable only among treatments reimbursed under medical benefit.

^c^ Concurrent medication use was defined as any MAUD (i.e., XR‐NTX, oral naltrexone, acamprosate, disulfiram) record during time on index.

^d^ Treatment discontinuation was defined as no subsequent record of index treatment in the 45‐day period following the previous record. The date of discontinuation was assigned as the end of days of supply.

^e^ If days of supply was missing, for XR‐NTX, it was imputed as 28 days, and for oral naltrexone, acamprosate, and disulfiram, it was imputed as 30 days (including date of initiation).

^f^ Calculated among patients who discontinued the index treatment within the 1‐year follow‐up period.

^g^ PDC was calculated as the total number of days in period “covered”/number of days in period (i.e., 365 days). In this calculation, each day is determined to be “covered” if medication is available for that day based on the days' supply.

**Figure 2:**
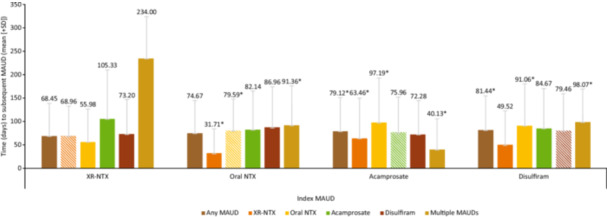
Mean (+SD) time to subsequent treatment following index treatment. MAUD, medications for alcohol use disorder; SD, standard deviation; XR‐NTX, extended‐release naltrexone. Striped bars represent re‐initiation of the index MAUD. Error bars represent the upper portion of the SD. *p‐Value < .05 for comparison with XR‐NTX.

The mean PDC for the XR‐NTX cohort was 0.34, compared with the oral naltrexone cohort (0.29), the acamprosate cohort (0.26), and the disulfiram cohort (0.29; all *p *< .01) (Table 2). In each cohort, the most frequently observed treatment received after the index treatment discontinuation was re‐initiation of the corresponding index treatment. In the XR‐NTX cohort, 28.3% of patients re‐initiated treatment with XR‐NTX after index XR‐NTX discontinuation, with a median time to re‐initiation of 38 days; 38.9%–42.9% of patients taking oral MAUDs re‐initiated treatment with the same MAUD, with a median time to re‐initiation of 51–53 days (Supplementary Information S1: Table 1). Fewer than half (45.9%) of all patients in the XR‐NTX cohort re‐initiated another FDA‐approved MAUD after discontinuation of the index treatment during the 1‐year follow‐up period, whereas a slightly higher proportion of patients in the other cohorts re‐initiated another MAUD after index treatment discontinuation (oral naltrexone: 52.0%; acamprosate: 52.2%; disulfiram: 53.6%; all *p* < .05*)*. The shortest time to re‐initiation of a MAUD occurred in patients who initiated XR‐NTX after discontinuing oral naltrexone (31.71 [SD 52.50] days; Figure 2).

### Healthcare resource utilization

**Figure 3:**
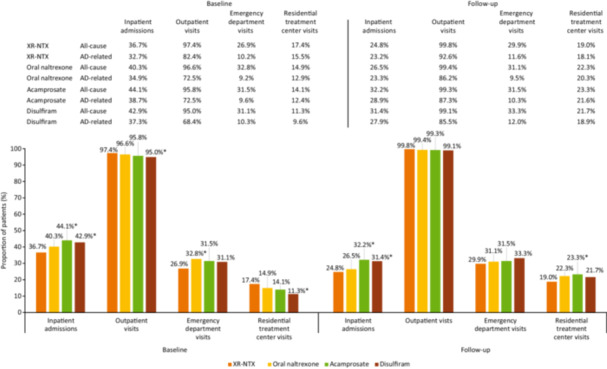
Proportion of patients with visits/admissions during baseline and follow‐up periods among MAUD cohorts. MAUD, medications for alcohol use disorder; XR‐NTX, extended‐release naltrexone. *p‐Value < .05 for comparison with XR‐NTX.

Compared with the baseline period, the proportion of patients with at least one inpatient admission during the follow‐up period was lower across all cohorts (XR‐NTX: 36.7% baseline vs. 24.8% follow‐up; oral naltrexone: 40.3% vs. 26.5%; acamprosate: 44.1% vs. 32.2%; disulfiram: 42.9% vs. 31.4%) (Figure 3).

Compared with the baseline period, the mean number of inpatient admissions per patient decreased for all MAUDs during the 1‐year follow‐up period (XR‐NTX: 0.48 vs. 0.42; oral naltrexone: 0.58 vs. 0.47; acamprosate: 0.67 vs. 0.60; disulfiram: 0.63 vs. 0.57) (Supplementary Information S1: Figure 2). The mean number of outpatient visits per patient increased across all cohorts during the follow‐up period (XR‐NTX: 20.0 vs. 36.0; oral naltrexone: 19.0 vs. 30.0; acamprosate: 19.0 vs. 31.0; disulfiram: 17.0 vs. 29.0).

## DISCUSSION

Veterans comprise a demographic more susceptible to AUD than the general population, and assessing the health economic impact and associated outcomes of treatment for AUD in this population is particularly critical.^14^, ^22^, ^23^ This study is among the first to provide insights and describe clinical characteristics, treatment patterns, and HCRU in VHA patients nationally who initiated MAUDs in a real‐world, coordinated care setting when all FDA‐approved MAUDs were listed on the VHA formulary. We observed 2 notable consequences of this policy decision. First, the overall use of MAUD increased during our study period. Second, these findings support the current VA guidelines that recommend the use of XR‐NTX among patients with AUD when medication adherence is a concern.^16^

The use of MAUDs among veterans with AUD has increased over time; however, most patients who could benefit from MAUDs continue not to receive them during treatment despite clinical guidelines supporting their use. For example, Harris et al. reported that 3.4% of VHA patients with AUD received a MAUD in 2009; this number increased to 5.8% in 2013 in a subsequent analysis.^12^, ^20^ In a single‐center study by Stewart et al., 9.5% of veterans with AUD between 2015 and 2018 received a MAUD.^24^ Our study found that 12.4% of veterans with AUD received a MAUD, even after employing more stringent inclusion criteria (e.g., required regular use of VHA system, no prior diagnosis of opioid use disorder). The higher observed rates of MAUD use in our study, as well as in the study by Stewart et al., relative to earlier VHA studies may reflect the removal of formulary barriers to providing MAUDs for veterans with AUD. Although this trend is encouraging, we simultaneously observed a >8‐month delay between AUD diagnosis and MAUD initiation, suggesting opportunities still remain to reduce delays in treatment.

Persistence with MAUD treatment is generally low,^11^, ^19^, ^25^ and our findings may reflect challenges to treatment adherence, including highly prevalent comorbid mental health and other substance use disorders in the VA population.^11^ Many veterans live in rural areas and face additional geographic barriers to accessing care that may affect treatment persistence.^22^ In this study, XR‐NTX was associated with higher adherence compared with oral MAUDs, as measured by PDC over a 1‐year follow‐up period, and was generally consistent with other retrospective studies conducted in veteran and nonveteran populations.^11^, ^19^, ^20^, ^25^, ^26^ One retrospective chart review of 715 patients with 807 medication trials from a single Veterans Integrated Service Network region found mean adherence, defined as the proportion of days the medication was available over a 6‐month period, was higher for veterans who received XR‐NTX (54.6%) versus oral MAUDs (41.3% for disulfiram, 44.7% for acamprosate, 49.8% for oral naltrexone).^11^ A larger study of patients with AUD across 129 VHA facilities found similar adherence to XR‐NTX, oral naltrexone, and disulfiram, all of which showed better adherence than to acamprosate.^20^ Adherence in that study was measured by PDC in the first 6 months after MAUD initiation. A chart review of 150 veterans with AUD found adherence during the first year of treatment was greater for XR‐NTX than for disulfiram and oral naltrexone at multiple timepoints, whereas PDC for XR‐NTX was numerically higher than, but not significantly different from, that for acamprosate.^24^ Comparative differences in adherence to MAUDs between our study and the published literature may reflect differences in baseline AUD severity, geographical differences, sample sizes, the timeframe over which PDC was calculated, or changes in MAUD management over time. The relatively higher adherence to treatment in patients receiving XR‐NTX compared with other MAUDs (which may be partly due to the reduced dosing frequency relative to other MAUDs) reported in these studies supports the VA guidelines recommendation to administer XR‐NTX when medication adherence is a concern.^16^

A retrospective cohort study in a nonveteran population found that patients taking oral MAUDs were 27%–49% more likely to discontinue treatment during 6 months of follow‐up compared with patients treated with XR‐NTX.^19^ This observation is consistent with the current study's findings that a significantly lower proportion of patients treated with XR‐NTX discontinued treatment during the 1‐year follow‐up period, versus patients treated with oral MAUDs (94.7% for XR‐NTX vs. 99.4% for oral NTX, 99.6% for acamprosate, and 99.4% for disulfiram; *p* < .001 for all comparisons between XR‐NTX and individual oral MAUDs).

In this study, the time to re‐initiation of the same MAUD was short for patients taking XR‐NTX (median 38 days), whereas the time to re‐initiation was slightly longer for the other MAUDs in this study (median 51–53 days). The study by Harris et al. found that the proportion of individuals who reached 90 days without a gap in medication possession was lowest (9.4%) among veterans receiving acamprosate and highest among those receiving XR‐NTX (20.2%).^20^ The shorter time to re‐initiating XR‐NTX may reflect that those patients are missing fewer doses than the patients taking other MAUDs. In addition, the current study used a gap of 45 days from the date of XR‐NTX receipt to define discontinuation. As a result, compared with studies that used larger gaps to define discontinuation, the current study may overestimate discontinuation by misclassifying patients between treatment episodes as having discontinued treatment. Many patients are inconsistently adherent to MAUD; thus, an apparent discontinuation of medication may simply represent a gap between treatment episodes with the same MAUD.^27^

Few studies have assessed HCRU specifically among veterans following initiation of pharmacotherapy for AUD, all of which were limited to XR‐NTX.^28^, ^29^ For example, Harris et al. reported a significant reduction in detoxification admissions after XR‐NTX initiation among VHA patients with AUDs, the majority of whom had received other MAUDs previously.^29^ Similarly, Chang et al. found that veterans with AUD had fewer psychiatric inpatient detoxification admissions in the year following XR‐NTX initiation when compared with the year before.^28^ We observed fewer hospitalizations and more outpatient visits among veterans in our study following initiation with any MAUD during this period.

### Limitations

This study should be considered within the context of certain limitations. As a retrospective study of healthcare claims data, diagnostic, prescription, and procedure codes were recorded for administrative purposes and may be subject to coding errors or omissions. The codes for behavioral or other nonpharmacological treatments received were not analyzed. Receipt of such treatments would likely have been similar across medications because psychosocial support is guideline‐recommended for all patients with AUDs^16^; however, these treatments could have affected outcomes, including the time to initiation of MAUD. Determining the severity of AUD is difficult, and the first observed moderate‐to‐severe AUD diagnosis during our study period may not have been the patient's first AUD diagnosis. Similarly, while we are selecting the patient's first MAUD, it is possible that this is not the first line of therapy, although exclusion criteria were used to mitigate this possibility (i.e., patients were excluded if they received MAUDs before the index date). The generalizability of this study's results to the entire US population may be limited, as the study sample consisted of veterans who received care within the VHA system, and the characteristics of patients in this study, including the large percentage of male patients and the number and types of comorbidities, differ from those of the US general population. For example, over 90% of veterans are screened annually for high‐risk alcohol use in VHA primary care.^30^ Most patients with moderate‐to‐severe AUD in the study did not receive a MAUD and were not included in the analyses. Comparisons with this cohort would be expected to increase the risk of bias from unbalanced patient characteristics; however, this may present an opportunity for future research. Patients included in this study were required to have regular use of the VHA system; however, it is possible that patients received care outside of the VHA system. Thus, some of the study outcomes may be underestimated. Finally, the study period concluded in 2019 and may not reflect more recent changes in MAUD utilization in the VA.

In this study, adherence was estimated based on refills and days' supply. For oral MAUDs, patients may not have used their entire days' supply. As a result, oral MAUD adherence was likely overestimated in our study. As a long‐acting injectable medication, XR‐NTX was supplied for the full 28‐day dosing interval at the time of administration. However, adherence to XR‐NTX may have been underestimated because administrations of XR‐NTX provided under the manufacturer's hospital‐based inpatient free trial program were not captured in the VHA data (i.e., patients who received only one dose of XR‐NTX through the manufacturer's program were excluded from this analysis).

This study aimed to provide a descriptive summary and did not control for differences in baseline characteristics among patients receiving XR‐NTX versus oral naltrexone, acamprosate, or disulfiram. Thus, the possibility remains that potential baseline differences could explain the improved adherence with XR‐NTX we observed. As XR‐NTX is recommended for patients with adherence concerns, this cohort may differ systematically from patients who received oral MAUDs. The sample sizes for XR‐NTX, acamprosate, and disulfiram were small relative to the oral naltrexone cohort, which may indicate that physicians are more likely to treat moderate‐to‐severe AUD with oral naltrexone first; thus, those not treated with oral naltrexone may be systemically different. As a result, caution should be used when drawing conclusions from the comparative analyses in this study. Similarly, we did not consider whether the observed decreases in inpatient use and increases in outpatient use varied by index MAUD. We note that these shifts only occurred for all MAUDs in the study. Although there was no pre‐specified hypothesis testing, the nominal *p* values based on the comparisons may be of value for prioritizing hypotheses for future research.

Finally, the VA/DoD guidelines also recommend the use of gabapentin and topiramate, which are not indicated for the treatment of AUD. Claims analyses cannot confirm the use of these medications for AUD, as opposed to other indications. As a result, examination of MAUDs was limited to FDA‐approved MAUDs.

## CONCLUSIONS

This retrospective, descriptive study provides important insights about real‐world outcomes associated with the increased use of MAUDs in the VA. First, the time on treatment was improved among veterans with AUD who initiated XR‐NTX relative to other MAUDs. Although discontinuation of initial MAUD treatment was common, the time to discontinuation was longest for XR‐NTX relative to other MAUDs, and many patients subsequently resumed treatment with the same initial treatment or a different MAUD. Second, treatment with MAUDs was associated with a decrease in the use of inpatient healthcare and an increase in outpatient visits. This shift in utilization may potentially reduce the use of more costly acute care and strengthen patient engagement, an important prognostic indicator for improved, long‐term outcomes. The insights gained from this study may assist healthcare decision‐makers with identifying means to improve medication adherence among patients with AUD and may provide insights that can prompt further research. Improving adherence can ultimately improve long‐term clinical and health economic outcomes and reduce the burden of illness associated with AUD. Further studies examining factors affecting adherence to and persistence with MAUDs, the impact on health outcomes of oral versus injection treatments, and the shift in HCRU from inpatient to outpatient care are warranted.

## CONFLICT OF INTEREST STATEMENT

R.G. is an employee of Alkermes, Inc. T.L.K., A.K.O., M.A.S., and J.L. were employees of Alkermes, Inc. during the conduct of the study. E.S., A.L., E.E.C., and S.S. are employees of Analysis Group, Inc., a consulting firm that received funding from the study's sponsor to conduct this study. K.W. is a member of the Alcohol Clinical Trials Initiative (ACTIVE) Workgroup, which has been supported previously, but not in the past 36 months, by Abbott/AbbVie, Amygdala Neurosciences, Arbor Pharmaceuticals, GSK, Indivior, Janssen, Lilly, Pfizer, and Schering Plough, but in the past 36 months, its activities were supported by Alkermes, Dicerna, Ethypharm, Lundbeck, Mitsubishi, and Otsuka. K.W. is also on the Scientific Advisory Board for Pear Therapeutics. S.L. was an employee of Southeast Louisiana Veterans Health Care System; Tulane University, who had no conflict of interest. The author(s), alone, is(are) responsible for the content and writing of this paper.

## Supporting information

Supplementary information.
