# Supplementary material for: Treatment patterns and healthcare resource use among veterans initiating medication for incident moderate‐to‐severe alcohol use disorder
Source: Am J Addict. 2025 May 12;34(5):536–46. doi: 10.1111/ajad.70036 (PMC12418735; doi:10.1111/ajad.70036)
Supplement: Supplementary file 1 — Supplementary information. [file AJAD-34-536-s001.docx]

**SUPPLEMENTAL MATERIALS**

**Supplemental Table 1**. Time to treatment re-initiation following discontinuation.

|  |  | **XR-NTX** | **Oral naltrexone** | ***p*-Value^a^** | **Acamprosate** | ***p*-Value^a^** | **Disulfiram** | ***p*-Value^a^** |
| --- | --- | --- | --- | --- | --- | --- | --- | --- |
|  |  | ***N* = 431** | ***N* = 25,082** |  | ***N* = 3,756** |  | ***N* = 2,115** |  |
| **First treatment initiated subsequent to index treatment discontinuation^b^** | |  |  |  |  |  |  |  |
| Any treatment, n (%) | | 198 (45.9) | 13,034 (52.0) | **<.05** | 1,960 (52.2) | **<.05** | 1,134 (53.6) | **<.01** |
| Days to any treatment initiation from index treatment discontinuation, mean ± SD [median] | | 68.45 ± 69.96 [37] | 74.67 ± 69.66 [48] | .213 | 79.12 ± 72.21 [50] | **<.05** | 81.44 ± 73.27 [53] | **<.05** |
| XR-NTX only, n (%) | | 122 (28.3) | 1,423 (5.7) | **<.001** | 41 (1.1) | **<.001** | 31 (1.5) | **<.001** |
| Days to XR-NTX initiation from index treatment discontinuation, mean ± SD [median] | | 68.96 ± 63.12 [38] | 31.71 ± 52.50 [14] | **<.001** | 63.46 ± 85.63 [19] | .707 | 49.52 ± 72.21 [21] | .139 |
| Oral naltrexone only, n (%) | | 60 (13.9) | 10,752 (42.9) | **<.001** | 336 (8.9) | **<.01** | 236 (11.2) | .103 |
| Days to oral naltrexone initiation from index treatment discontinuation, mean ± SD [median] | | 55.98 ± 70.07 [26] | 79.59 ± 68.05 [52] | **<.01** | 97.19 ± 95.53 [62] | **<.01** | 91.06 ± 88.75 [57] | **<.01** |
| Acamprosate only, n (%) | | 9 (2.1) | 561 (2.2) | .836 | 1,535 (40.9) | **<.001** | 30 (1.4) | .302 |
| Days to acamprosate initiation from index treatment discontinuation, mean ± SD [median] | | 105.33 ± 99.36 [78] | 82.14 ± 85.72 [48] | .422 | 75.96 ± 64.53 [51] | .402 | 84.67 ± 90.45 [48] | .560 |
| Disulfiram only, n (%) | | 5 (1.2) | 229 (0.9) | .603 | 40 (1.1) | .805 | 822 (38.9) | **<.001** |
| Days to disulfiram initiation from index treatment discontinuation, mean ± SD [median] | | 73.20 ± 85.05 [35] | 86.96 ± 92.60 [44] | .742 | 72.28 ± 87.54 [28] | .982 | 79.46 ± 67.20 [53] | .836 |
| Multiple MAUDs, n (%) | | 2 (0.5) | 69 (0.3) | .338 | 8 (0.2) | .275 | 15 (0.7) | .753 |
| Days to MAUD initiation from index treatment discontinuation, mean ± SD [median] | | 234.00 ± 89.10 [234] | 91.36 ± 84.50 [56] | **<.05** | 40.13 ± 64.61 [22] | **<.01** | 98.07 ± 70.39 [78] | **<.05** |

Abbreviations: MAUD, medication for alcohol use disorder; SD, standard deviation; XR-NTX, extended-release naltrexone.
^a^Statistical comparisons were conducted between XR-NTX and other MAUD cohorts using *t*-tests for continuous variables and chi-square tests for categorical variables. A *p*-value <.05 was considered statistically significant.
^b^Calculated among patients who discontinued index treatment within the 1-year follow-up period.

**Supplemental Figure 1.** Study design diagram.

**
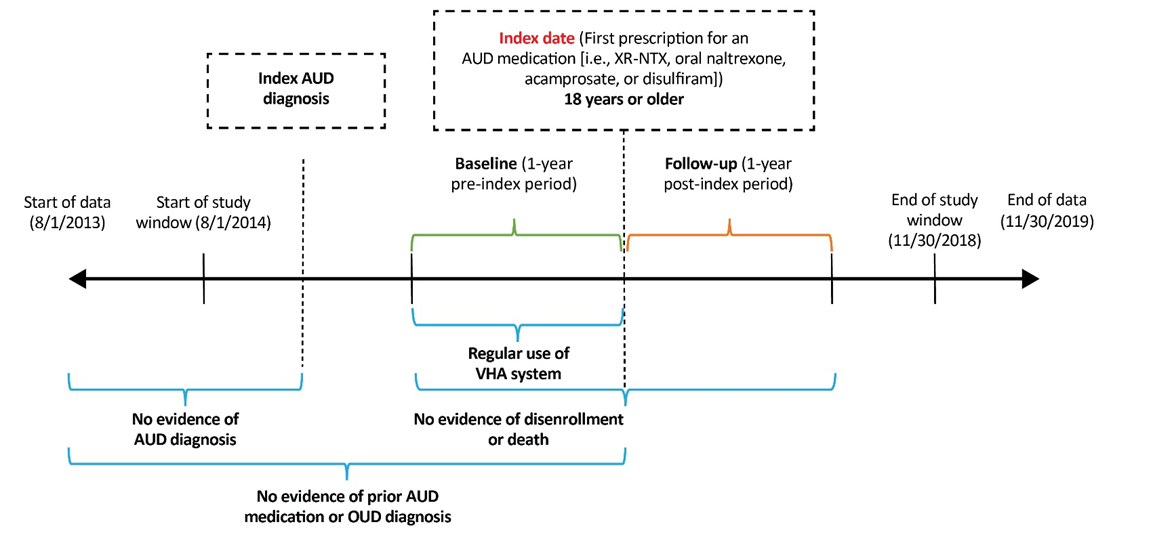
**

Abbreviations: AUD, alcohol use disorder; OUD, opioid use disorder; VHA, Veterans Health Administration; XR-NTX, extended-release naltrexone.

**Supplemental Figure 2.** Number of visits/admissions per patient during baseline and follow-up periods among MAUD cohorts.

**
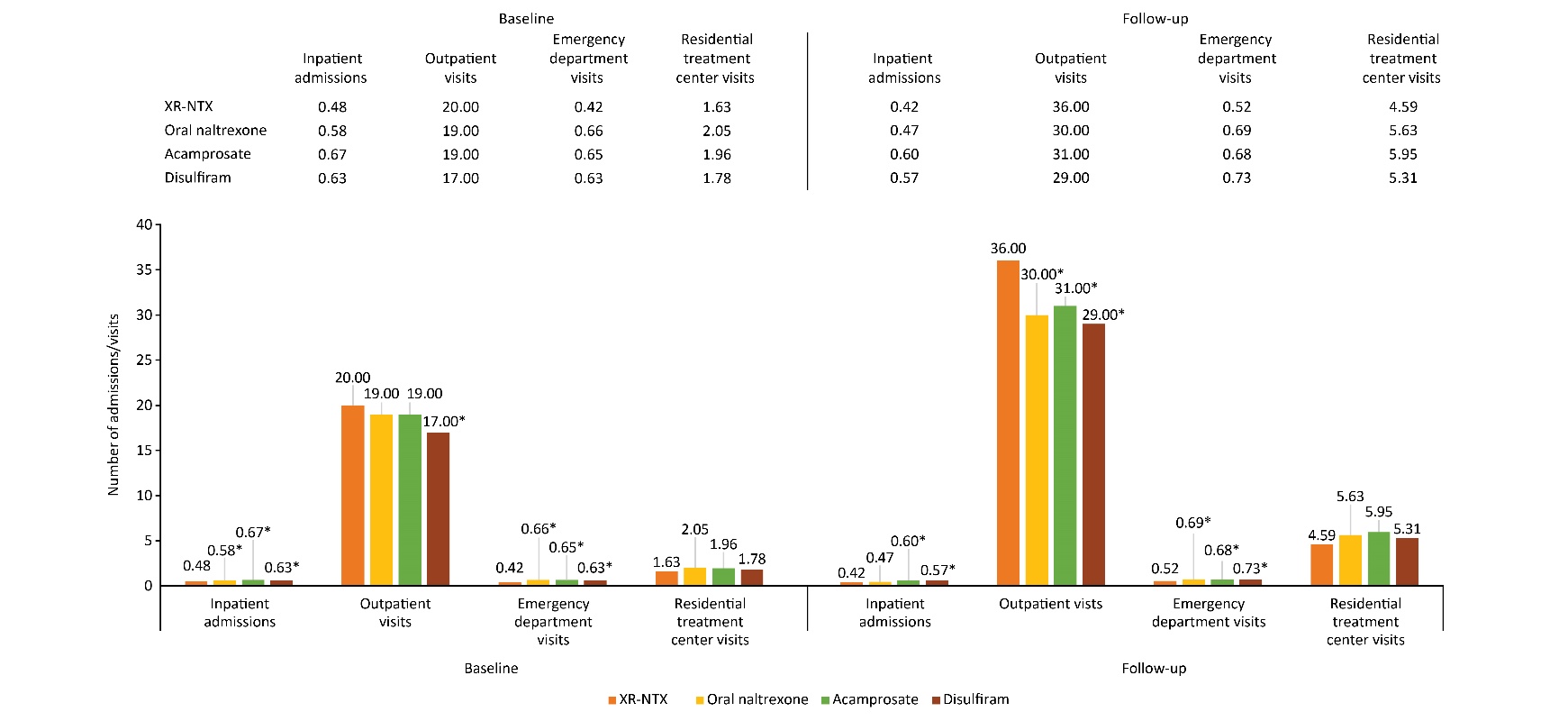
**

Abbreviations: MAUD, medications for alcohol use disorder; XR-NTX, extended-release naltrexone.

**Note:** *p-Value <.05 for comparison with XR-NTX.
